# From QUOROM to PRISMA: A Survey of High-Impact Medical Journals' Instructions to Authors and a Review of Systematic Reviews in Anesthesia Literature

**DOI:** 10.1371/journal.pone.0027611

**Published:** 2011-11-16

**Authors:** Kun-ming Tao, Xiao-qian Li, Qing-hui Zhou, David Moher, Chang-quan Ling, Wei-feng Yu

**Affiliations:** 1 Department of Anesthesiology, Eastern Hepatobiliary Surgical Hospital, Second Military Medical University, Shanghai, China; 2 Department of Traditional Chinese Medicine, Changhai Hospital, Second Military Medical University, Shanghai, China; 3 Clinical Epidemiology Program, Ottawa Hospital Research Institute, Ottawa, Canada; University of York, United Kingdom

## Abstract

**Background:**

The PRISMA (*P*referred *R*eporting *I*tems of *S*ystematic reviews and *M*eta-*A*nalyses) Statement was published to help authors improve how they report systematic reviews. It is unknown how many journals mention PRISMA in their instructions to authors, or whether stronger journal language regarding use of PRISMA improves author compliance.

**Methodology/Principal Findings:**

An Internet-based investigation examined the extent to which 146 leading medical journals have incorporated the PRISMA Statement into their instructions to authors. Results were analyzed using descriptive statistics. Also, systematic reviews published in the leading anesthesiology journals and the QUOROM (*QU*ality *O*f *R*eporting *O*f *M*eta-analyses) Statement were used to explore the hypothesis that indicating compliance with the QUOROM Statement in the manuscript is associated with improved compliance with the reporting guideline. In a sample of 146 journals publishing systematic reviews, the PRISMA Statement was referred to in the instructions to authors for 27% (40/146) of journals; more often in general and internal medicine journals (7/14; 50%) than in specialty medicine journals (33/132; 25%). In the second part of the study, 13 systematic reviews published in the leading anesthesiology journals in 2008 were included for appraisal. Mention of the QUOROM Statement in the manuscript was associated with higher compliance with the QUOROM checklist (*P* = 0.022).

**Conclusions/Significance:**

Most of the leading medical journals used ambiguous language regarding what was expected of authors. Further improvement on quality of reporting of systematic reviews may entail journals clearly informing authors of their requirements. Stronger directions, such as requiring an indication of adherence to a research quality of reporting statement in the manuscript, may improve reporting and utility of systematic reviews.

## Introduction

In 1999, to address suboptimal reporting of meta-analyses, an international group published the QUOROM (*QU*ality *O*f *R*eporting *O*f *M*eta-analyses) Statement [1]. Many journal editors and authors, including those involved in the Cochrane Collaboration, then pursued compliance with the QUOROM checklist to ensure that authors reported transparently what they did (methods) and found (results) [2].

In 2009, the QUOROM Statement was updated to address several conceptual, methodological and practical advances, and was renamed PRISMA (*P*referred *R*eporting *I*tems of *S*ystematic reviews and *M*eta-*A*nalyses) [3]. Although the PRISMA Group advised that PRISMA should replace QUOROM for those journals that endorsed QUOROM, it was unclear how many journal websites reference the PRISMA Statement in their instructions to authors. Thus in this study, the extent to which leading medical journals have incorporated the PRISMA Statement into their instructions to authors was evaluated.

In the course of the investigation, some strong requirements regarding systematic reviews were found among journals' instructions to authors; one of them is that authors should indicate in the manuscript that relevant reporting statements have been followed. Hence, in the second part of this study, the hypothesis that referencing a reporting statement in a systematic review manuscript would correspond with improved compliance with the guidance was tested, using systematic reviews published in leading anesthesiology journals. Since the PRISMA Statement was released just two years previously and preparation of a systematic review may be a lengthy process [4], it was conceivable that few reviews may indicate compliance with the PRISMA Statement. Therefore, we tested the hypothesis by examining reference to and compliance with the QUOROM Statement, in systematic reviews published in 2008.

## Methods

### Identification of High Impact Journals for PRISMA Investigation

**Figure 1:**
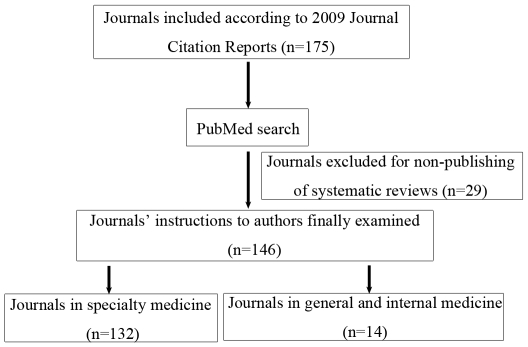
Flow diagram of journals included in this study. By using the 2009 citation impact factors, the top five journals from each of the 34 medical categories, and the top 15 journals from general and internal medicine categories were identified (n = 175). A pre-defined PubMed search strategy without time limits was used to identify whether a journal had published systematic reviews or not. The final sample of 146 journals that published systematic reviews was included for investigation of requirements for PRISMA Statement in their instructions to authors.

For investigation of requirements for PRISMA in Authors' Instructions, by referencing to Altman's study [5], the top five journals from each of the 34 medical categories, and the top 15 journals from general and internal medicine categories were identified using 2009 citation impact factors (Journal Citation Reports of Thomson Reuters). Eight journals were classified in two or three categories. Journals that did not publish systematic reviews were identified using journal-specific PubMed searches, using a recognized search strategy, without time limits [6]. The final sample of 146 journals that published systematic reviews was obtained after examining 175 journals (Figure 1).

### Assessing Endorsement

Between February 21^st^, 2011 and July 15^th^, 2011, two assessors (Tao KM and Li XQ) independently examined the Instructions to Authors section of the website of each of the 146 journals, and extracted any text mentioning “PRISMA” or “QUOROM.” For those journals that endorsed the PRISMA Statement, all reference sources such as “PRISMA” or “EQUATOR” (*E*nhancing the *QUA*lity and *T*ransparency *O*f health *R*esearch) web addresses, as well as requirements regarding the PRISMA or QUOROM Statement were also recorded.

### Critical Appraisal of Anesthesia Systematic Reviews

Systematic reviews published in the top five anesthesiology journals in 2008 were sought, using a pre-defined strategy (**Document S1**) [6]. As the QUOROM Statement mainly focuses on the quality of reporting meta-analyses of randomized controlled trials [1], only systematic reviews of randomized controlled trials were included. Two authors (Tao KM and Li XQ) independently appraised each systematic review for compliance with each QUOROM checklist item. A third author (Yu WF) was consulted when consensus between the two reviewers could not be reached. For this study, a manuscript was considered to be compliant with any of the 18 specific items on the QUOROM checklist if over 50% of the requirements for the particular item were met. A score was calculated according to the number of items met by each manuscript [2]. The manuscripts under review were divided into two groups according to whether or not the QUOROM Statement was mentioned in the manuscript, and the scores of the manuscripts in the two groups were compared. Finally, the association between the manuscript score and the length of the manuscript (number of pages) was evaluated.

### Statistical Analysis

Agreement between the two reviewers on the QUOROM checklist items were assessed using the Cohen κ coefficient. Scores of the QUOROM Statement items indicated in the manuscripts were compared using the Mann-Whitney U test. Potential correlation between the QUOROM scores and length of the manuscript were investigated using both Pearson and Spearman correlation coefficients. Analyses were carried out using SPSS version 16.0.

## Results

### Assessing Endorsement

The PRISMA Statement was referred to in the instructions to authors of 40 (40/146; 27%) journals; more often in general and internal medicine journals (7/14; 50%) than in those for other specialties (33/132; 25%). Of the 40 journals, 27 gave the PRISMA Statement web address, six gave the EQUATOR web address, five cited the 2009 PRISMA Statement [3], [7], [8], one gave the CONSORT web address, while the other one did not reference anything. Of the 40 journals referring to the PRISMA Statement, 28 asked or encouraged authors to report systematic reviews in accordance with the PRISMA Statement; 11 required or encouraged authors to submit the PRISMA flow diagram and/or checklist with the manuscript; the other one journal asked authors to indicate in the manuscript that they had complied with the PRISMA Statement. Two (2/146; 1%) journals still referred to the QUOROM Statement by the 1999 publication of the QUOROM Statement.

### Critical Appraisal of Anesthesia Systematic Reviews

**Figure 2:**
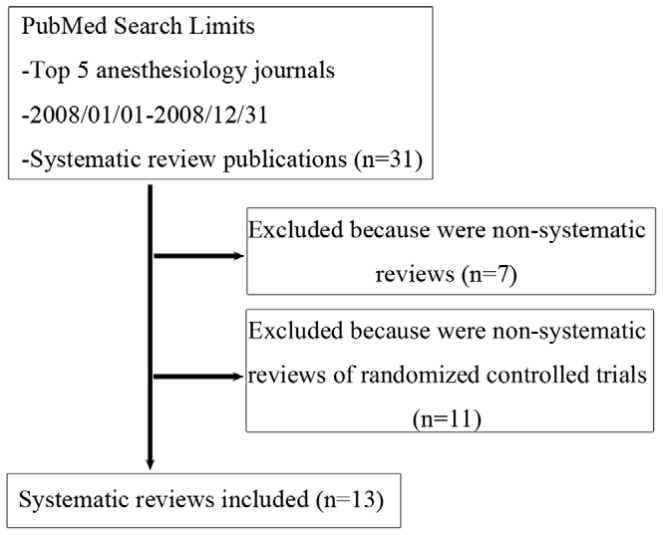
Flow diagram of systematic reviews included in this study. Systematic reviews published in the top five anesthesiology journals in 2008 were sought by using a pre-defined PubMed search strategy (n = 31). Thirteen systematic reviews of randomized controlled trials were finally included for appraising their compliance with QUOROM checklist.

Thirteen systematic reviews published in the top five anesthesiology journals were included for appraisal (Figure 2) [9]–[21]. And these systematic reviews are all from journals that did not reference to the QUOROM Statement in their instructions to authors. Agreement between assessors regarding compliance with the QUOROM items was good (219/234, κ = 0.78, 95% confidence interval 0.68 to 0.89). The median score of compliance with the QUOROM checklist was 15 (i.e. 15 of 18 items on the QUOROM checklist were included in the manuscript) (Table 1). No relationship was identified between the number of published pages and the overall QUOROM score (Pearson *r* = −0.212, *P* = 0.486; Spearman ρ = −0.048, *P* = 0.876); however, reference to the QUOROM Statement in the manuscript was associated with a higher QUOROM score (*P* = 0.022).

**Table 1:** Compliance of Included Studies with the QUOROM Checklist*.

CharacteristicCitation number	Compliance												
	9	10	11	12	13	14	15	16	17	18	19	20	21
Identifying title	+	—	+	—	—	—	—	+	—	—	+	—	—
Abstract													
Structured	+	+	+	+	+	—	—	—	—	—	—	+	—
Objectives	+	+	+	+	+	+	+	+	+	+	+	+	+
Data sources	+	—	—	—	+	+	+	+	—	+	—	—	+
Review methods	—	—	+	—	+	+	+	+	—	—	—	+	—
Results	+	+	+	+	+	+	+	+	+	+	+	+	+
Conclusion	+	+	+	+	+	+	+	+	+	+	+	+	+
Introduction	+	+	+	+	+	+	+	+	+	+	+	+	+
Methods													
Searching	+	+	+	+	+	+	+	+	+	+	+	+	+
Selection	+	+	+	+	+	+	+	+	+	+	+	+	+
Validity assessment	—	—	+	+	+	+	+	+	+	+	+	+	+
Data abstraction	+	—	+	+	+	+	+	+	+	+	+	+	+
Study characteristics	+	+	+	+	+	+	+	+	+	+	+	+	+
Quantitative data synthesis	+	+	+	+	+	+	+	+	+	+	+	+	+
Results													
Trial flow	+	—	+	—	—	—	+	+	+	+	—	—	+
Study characteristics	+	+	+	+	+	+	+	+	+	+	+	+	+
Quantitative data synthesis	+	+	+	+	+	+	+	+	+	+	+	+	+
Discussion	+	+	+	+	+	+	+	+	+	+	+	+	+
Total QUOROM score	16	12	17	14	16	15	16	17	14	15	14	15	15
Number of pages	14	13	8	7	8	11	8	10	8	8	11	6	9
Explicit quotation of the QUOROM Statement by authors	+	—	+	—	+	—	—	+	—	+	—	—	+

+: more than 50% of requirements satisfied; —: non-compliance.

*compliance with the QUOROM items was assessed by two independent reviewers.

## Discussion

Among 146 high-impact medical journals, in 2011 half of general and internal medicine publications and a quarter of other specialty journals referred to the 2009 PRISMA Statement in their instructions to authors; 1% still referenced the QUOROM Statement. Most journals used ambiguous language, not stating explicitly what was expected from the contributors, and only 12 journals encouraged or required authors to submit the PRISMA checklist or to state in the manuscript that PRISMA was followed. Anesthesiology systematic reviews published in 2008 on average reported 15 out of 18 QUOROM items, with higher numbers reported in reviews explicitly mentioning QUOROM.

The quality of reporting of systematic reviews is not optimal, yet the clarity of reporting greatly affects the value of systematic review [22], [23]. Based on the QUOROM Statement, the PRISMA Statement was developed to further improve the quality of reporting of systematic reviews.

Requirements noted in instructions to authors of systematic reviews to adhere to the PRISMA Statement are somewhat less common than previously reported regarding use of the CONSORT (*C*onsolidated *S*tandards *O*f *R*eporting *T*rials) Statement [24]. In 2008, Hopewell *et al.* found that CONSORT was endorsed in instructions to authors by 38% of 165 high-impact factor medical journals [24]. As CONSORT was developed 3 years earlier than QUOROM [25], the difference seems plausible. Moreover, just as found presently for the PRISMA Statement, the proportion of journals endorsing the CONSORT Statement was lower in specialty medicine journals than in general and internal medicine journals [5]. Indeed, in some specialty medicine categories in the present study, none of the top five journals' instructions to authors refer to the PRISMA Statement. Although the reasons are not clear, this may be reflected in the result that methodology of meta-analyses published in general medicine journals was superior to those published in specialty medicine journals [26].

Referring to the PRISMA Statement or including the PRISMA web address in a journal's instructions to authors is a good way to remind authors of the importance of transparent reporting of systematic reviews [3]. However, its effectiveness will be diminished by ambiguous statements in journals' instructions to authors. This study found that most journals supporting PRISMA did not state their expectations for authors clearly, and few required authors to submit the PRISMA checklist. In the form of a checklist, the PRISMA group provides advice on how to report research methods and findings, and the checklist specifies a minimum set of items required for a clear and transparent reporting of systematic reviews [8]. As there is growing evidence that use of a checklist is beneficial [27], using PRISMA checklists in writing and peer reviewing of systematic reviews should be encouraged.

In this study, we also found that some journals linked the PRISMA statement to the EQUATOR Network website, which is an international initiative focusing on dissemination of the basic principles of responsible research reporting and the wider implementation of reporting guidelines [27]. The EQUATOR website provides up-to-date guidance on reporting, scientific writing and ethical conduct in research and publication for researchers, editors and peer reviews. It also provides resources for scientists who wish to develop further high-quality reporting guidelines [27]. As everyone involved in the process of research and its publication needs to participate in the course of practicing accurate and transparent reporting of health research studies, it is encouraged that journals refer to the EQUATOR Network website in their instructions for authors.

In a previous study, the manuscript length was associated with higher compliance with the QUOROM Statement [2] ^,^ but this finding is not confirmed by the present study. Possible reason is that with the improvement of the quality of systematic reviews in medical journals year by year [28], shorter ones and those have methodological shortcomings are more likely to be rejected. Like Biondi-Zoccai's research result [2], although compliance with the 18 items on the QUOROM checklist was high in the present study, there is still room for considerable improvement in reporting of systematic reviews, in accordance with the guideline. Further improvements in quality of reporting may entail journals clearly informing authors of their requirements. The present study investigated whether a clear acknowledgement of the guideline, such as the authors' statement of compliance with QUOROM in the manuscript, affects effectiveness, and the result is positive. This suggests that indicating the reporting statement in the manuscript is not only a simple acknowledgement of the guideline, but that it may also enhance the awareness of the investigators, the reviewers and the journal editors of complete and transparent reporting of systematic reviews, and that this may promote the use of the PRISMA Statement.

There are also limitations in our study. First, examination of instructions to authors entailed review of electronic resources that may have changed after the investigation; however, no journal updated the instructions for authors during the study period. Second, investigation of systematic reviews was limited to anesthesiology journals. Systematic reviews of this specialty have been considered to be higher quality than reviews published in other specialty medicine journals [28], so the conclusion of this study should be treated cautiously. Finally, in light of the recent publication of PRISMA, this study evaluated systematic reviews according to the QUOROM rather than the PRISMA checklist. In a related matter, the QUOROM checklist was used to appraise the systematic reviews; it should be noted that the QUOROM checklist was not developed as a quality measurement tool and the validation of this application is unclear [2]. Further investigations into reporting quality of systematic reviews using PRISMA should be carried out, following longer acceptance and utilization of this Statement.

## Supporting Information

Document S1 **PubMed search strategy for identifying systematic reviews published in the top five anesthesiology journals in 2008.**
(DOC)
