# Supplementary material for: From QUOROM to PRISMA: A Survey of High-Impact Medical Journals' Instructions to Authors and a Review of Systematic Reviews in Anesthesia Literature
Source: PLoS One. 2011 Nov 16;6(11):e27611. doi: 10.1371/journal.pone.0027611 (PMC3217994; doi:10.1371/journal.pone.0027611)
Supplement: Document S1 — PubMed search strategy for identifying systematic reviews published in the top five anesthesiology journals in 2008. (DOC) [file pone.0027611.s001.doc]

PubMed Search

Five journals with the highest impact factors were selected from the “anesthesiology” category of the 2008 Journal Citation Reports (JCR) from Thomson Reuters. The ISSN (Print) was used to perform searches in PubMed.

- ((0304-3959) OR (0003-3022) OR (1090-3801) OR (0007-0912) OR (0749-8047))

AND

- ((meta-analysis [pt] OR meta-analysis [tw] OR metanalysis [tw]) OR ((review [pt] OR guideline [pt] OR consensus [ti] OR guideline* [ti] OR literature [ti] OR overview [ti] OR review [ti]) AND ((Cochrane [tw] OR Medline [tw] OR CINAHL [tw] OR (National [tw] AND Library [tw])) OR (handsearch* [tw] OR search* [tw] OR searching [tw]) AND (hand [tw] OR manual [tw] OR electronic [tw] OR bibliographi* [tw] OR database* OR (Cochrane [tw] OR Medline [tw] OR CINAHL [tw] OR (National [tw] AND Library [tw]))))) OR ((synthesis [ti] OR overview [ti] OR review [ti] OR survey [ti]) AND (systematic [ti] OR critical [ti] OR methodologic [ti] OR quantitative [ti] OR qualitative [ti] OR literature [ti] OR evidence [ti] OR evidence-based [ti]))) BUTNOT (case* [ti] OR report [ti] OR editorial [pt] OR comment [pt] OR letter [pt])

**Limits Activated:** Publication Date from 2008/01/01 to 2008/12/31
